# Central nociceptive sensitization vs. spinal cord training: opposing forms of plasticity that dictate function after complete spinal cord injury

**DOI:** 10.3389/fphys.2012.00396

**Published:** 2012-10-04

**Authors:** Adam R. Ferguson, J. Russell Huie, Eric D. Crown, James W. Grau

**Affiliations:** ^1^Department of Neurological Surgery, Brain and Spinal Injury Center, University of California San FranciscoSan Francisco, CA, USA; ^2^Abbott LaboratoriesChicago, IL, USA; ^3^Cellular and Behavioral Neuroscience, Department of Psychology, Texas A&M UniversityCollege Station, TX, USA

**Keywords:** pain, nociception, plasticity, spinal cord injury, spinal cord learning, recovery of function

## Abstract

The spinal cord demonstrates several forms of plasticity that resemble brain-dependent learning and memory. Among the most studied form of spinal plasticity is spinal memory for noxious (nociceptive) stimulation. Numerous papers have described central pain as a spinally-stored memory that enhances future responses to cutaneous stimulation. This phenomenon, known as central sensitization, has broad relevance to a range of pathological conditions. Work from the spinal cord injury (SCI) field indicates that the lumbar spinal cord demonstrates several other forms of plasticity, including formal learning and memory. After complete thoracic SCI, the lumbar spinal cord can be trained by delivering stimulation to the hindleg when the leg is extended. In the presence of this response-contingent stimulation the spinal cord rapidly learns to hold the leg in a flexed position, a centrally mediated effect that meets the formal criteria for instrumental (response-outcome) learning. Instrumental flexion training produces a central change in spinal plasticity that enables future spinal learning on both the ipsilateral and contralateral leg. However, if stimulation is given in a response-independent manner, the spinal cord develops central maladaptive plasticity that undermines future spinal learning on both legs. The present paper tests for interactions between spinal cord training and central nociceptive sensitization after complete spinal cord transection. We found that spinal training alters future central sensitization by intradermal formalin (24 h post-training). Conversely intradermal formalin impaired future spinal learning (24 h post-injection). Because formalin-induced central sensitization has been shown to involve NMDA receptor activation, we tested whether pre-treatment with NMDA would also affect spinal learning in manner similar to formalin. We found intrathecal NMDA impaired learning in a dose-dependent fashion, and that this effect endures for at least 24 h. These data provide strong evidence for an opposing relationship between nociceptive plasticity and use-dependent learning in the spinal cord. The present work has clinical implications given recent findings that adaptive spinal training improves recovery in humans with SCI. Nociception below the SCI may undermine this rehabilitation potential.

## Introduction

Research over the past 50 years has revealed the spinal cord to be surprisingly plastic. The spinal cord has been shown to support a number of simple forms of learning, including habituation and sensitization, as well as Pavlovian and instrumental conditioning (Thompson and Spencer, 1966; Fitzgerald and Thompson, 1967; Grau et al., 1998). This remarkable capacity for adaptability in response to stimuli has led researchers to investigate how spinal plasticity might be utilized to promote functional recovery after spinal cord injury (SCI). To this end, researchers have designed behavioral training programs to engage the inherent plasticity in spinal motor systems. Training on a treadmill has been shown to evoke locomotor activity, and induce weight-supported stepping in completely transected cats (Lovely et al., 1986; Barbeau and Rossignol, 1987; De Leon et al., 1998). Similar effects have been observed in a variety of species, and under varying conditions, demonstrating that multiple forms of locomotor training can induce adaptive alterations in spinal plasticity that improves recovery after SCI (Edgerton et al., 1992; Bregman et al., 1997; Raineteau and Schwab, 2001; Edgerton and Roy, 2009). In order for functional recovery to be successful, appropriate sensory feedback, including proprioceptive and cutaneous afferent input, is necessary (Bouyer et al., 2001; Bouyer and Rossignol, 2003). Unilateral deafferentation of spinally transected cats results in impaired locomotor recovery (Giuliani and Smith, 1987). Likewise, the disruption of even a small number of peripheral nerves prior to SCI can greatly limit adaptive locomotor recovery (Bouyer and Rossignol, 2003; Frigon and Rossignol, 2009). These findings highlight the essential role of afferent input in inducing plasticity within locomotor circuits. If the delicate interaction between sensory input and motor output is disturbed, recovery of spinal function can be severely impaired (Frigon and Rossignol, 2009).

These findings illustrate the plastic nature of the spinal cord, and highlight the importance of promoting adaptive spinal modifications in order to combat the deleterious effects of SCI.

The capacity for plasticity after SCI also creates an environment in which the spinal cord is vulnerable to maladaptive changes. Potentiation of the response to nociceptive afferent input in the superficial dorsal horn can produce lasting changes in pain reactivity. This phenomenon, known as central sensitization, may be a mechanism by which intractable neuropathic pain is induced (Woolf, 1983; Woolf and Salter, 2000). Interestingly, this effect bears a striking resemblance to long-term potentiation (LTP), an NMDA-mediated process that has been long-believed to be the neurobiological basis for learning and memory (Bliss and Lomo, 1973; Ikeda et al., 2003; Ji et al., 2003; Woolf, 2007). The similarity between LTP in the brain and central sensitization in the spinal cord has raised the possibility that nociceptive plasticity is akin to a form of learning, and lasting pain states are in essence a nociceptive memory (Sandkuhler et al., 2000; Ji et al., 2003; Crown et al., 2005, 2006, 2008).

From the evidence presented above, it is apparent that the spinal cord is capable of supporting a wide range of plastic processes that, depending on the type of stimulus, can either promote adaptive changes or exacerbate nociceptive activity. Despite our knowledge of these opposing processes, the relationships between spinal nociceptive memory and spinal memory for adaptive training are not well-understood. Yet, uncovering the interactions between these different forms of spinal neuroplasticity has clinical relevance for developing rehabilitative therapies that maximize beneficial recovery and minimize harmful side effects such as pain. To study spinal plasticity mechanistically we have used a simple model of instrumental learning in the isolated spinal cord (Buerger and Fennessy, 1970; Grau et al., 1998). Rats with complete spinal transections receive electrical stimulation to the tibialis anterior muscle of one hindlimb whenever that limb is extended (*controllable stimulation*). Rats learn over time to keep the limb flexed in order to reduce stimulation exposure, thus exhibiting a simple form of instrumental (response-outcome) learning. This training paradigm has been shown to produce a number of beneficial effects on spinal function (see Ferguson et al., 2012 and Grau et al., 2012, in this issue).

Conversely, if rats receive electrical stimulation of the tibialis anterior muscle that is independent of leg position (*uncontrollable stimulation*), they are unable to learn the instrumental response. Further, when these rats are later tested with controllable stimulation, they continue to exhibit a learning deficit, even if rats are tested on the contralateral limb (Joynes et al., 2003). This finding, along with a series of other pharmacological and physiological studies, indicates that prior exposure to uncontrollable stimulation does not simply produce a peripheral, motoric effect, but instead induces a lasting maladaptive alteration in spinal plasticity (Crown et al., 2002a; Baumbauer et al., 2009; for review see Grau et al., 2006). Interestingly, prior work indicates that uncontrollable stimulation also induces nociceptive plasticity (Ferguson et al., 2006; Huie et al., 2012a). Tests of tactile reactivity with von Frey filaments have shown that uncontrollable (yoked) stimulation can induce mechanical allodynia (Ferguson et al., 2006; Huie et al., 2012a). Interestingly, both the induction of the spinal learning response and the induction of the learning deficit have been shown to require NMDA activation, providing further mechanistic similarities between adaptive and nociceptive plasticity and learning in the spinal cord. To further elucidate whether uncontrollable stimulation induces spinal nociceptive activity, we have previously used electrophysiological and pharmacological methods to investigate the fiber types that are engaged by this stimulation regimen. Baumbauer et al. (2007), found that intrathecal blockade of the NK1 (substance P) receptor blocked the induction of a learning deficit normally produced by uncontrollable stimulation. Likewise, electrical stimulation of the sciatic nerve did not induce a learning impairment until shock intensity was increased to a level that engaged C-fibers (Baumbauer et al., 2008). While instrumental learning is not blocked by pre-treatment with a NK1 antagonist, the shock intensity needed to elicit an intermediate flexion force is well within the range that Baumbauer et al. (2008) found to elicit some C-fiber activity and a robust A-delta response. On the basis of these observations, we have suggested that the induction of the learning deficit requires C-fiber activity.

Prior work has also shown that spinal learning deficits can be induced by the peripheral administration of inflammatory agents known to induce central sensitization, such as carrageenan, and capsaicin (Ferguson et al., 2006; Hook et al., 2008). As with uncontrollable stimulation, peripheral inflammation induced a learning deficit that was observed when testing was administered on the contralateral limb, yielding further evidence for central nociceptive plasticity. Thus, using this simple behavioral model of spinal plasticity provides a mechanism to study both maladaptive nociceptive plasticity and adaptive alterations in spinal learning. The present study is designed to gain further insight into these opposing processes, by testing the interaction between spinal training and nociceptive plasticity. We first ask how spinal training history affects nociceptive plasticity, and then conversely, how nociceptive activity may alter the capacity for spinal learning.

We first assessed the capacity for an inflammatory agent known to induce central sensitization (intradermal formalin) to produce tactile hyper-reactivity in the spinally transected rat. We then tested what effect spinal training (with either controllable or uncontrollable stimulation) prior to formalin administration may have on nociceptive responding. To further assess the interaction between nociceptive plasticity and spinal learning, we then tested whether formalin administration is sufficient to undermine spinal learning, and whether this effect is centrally-mediated. Finally, given the findings that central sensitization is an NMDA receptor-mediated process (Dickenson and Sullivan, 1991), we tested whether direct central activation of NMDA receptors induces a spinal learning deficit.

## Materials and methods

We performed three independent experiments to investigate the relationship between spinal learning and nociceptive sensitization. In the first experiment we delivered three different spinal cord training procedures and then 24 h later delivered intradermal formalin or vehicle (3 training groups × 2 formalin conditions; *n* = 7/group; *N* = 42 total). We then evaluated tactile responsiveness (Figures 2–4). In a separate group of rats we delivered intradermal formalin and then evaluated spinal learning potential immediately as a function of formalin dose-response (Figure 5; *n* = 2–6/dose group; *N* = 17 total). In an independent replication of the most effective dose, we tested the effects of formalin on spinal learning 24 h later (*n* = 6/group, *N* = 12 total). Finally, in a third set of rats we delivered intrathecal NMDA at doses that are known to produce spontaneous nociception and tested spinal training potential 24 h later (*N* = 12/group; *N* = 48 total). The experimental designs for each study are depicted in Figures 2, 5A,C, and 6A. The specific procedures are described below.

### Animals

Subjects were adult (100–120 day old, *N* = 119) male Sprague-Dawley rats (Harlan, Houston, TX, USA). Rats were individually housed in an AAALAC-approved, temperature-controlled environment with *ad libitum* access to food and water. Rats were maintained on a 12 h light/dark cycle with experiments performed during the last 6 h of the light cycle. All experiments adhered to the NIH Guide and were approved by the Animal Care Committee at Texas A&M University.

### Spinal cord transection and intrathecal cannula insertion

Surgery was performed under pentobarbital anesthesia (50 mg/kg, i.p.). Rats were placed in a stereotaxic instrument and a small gauze “pillow” was placed under the chest to raise and support the area around the second thoracic vertebra (T2). After localizing T2 through the skin, a rostro-caudal incision was made and the muscles were blunt dissected to reveal the intervertebral space rostral to T2. Rongeurs were used to clear ligaments and expose the spinal cord in between T1 and T2 and the spinal cord was transected by cauterization. Gel foam was placed in the transection site. For intrathecal drugs delivery experiments, a cannula was implanted after spinal transection. The cannula consisted of 25 cm of PE-10 tubing fitted with a 0.23 mm (diameter) stainless steel guide wire (Small Parts, USA) that was threaded 9 cm caudally from T2 into the subarachnoid space between dura and white matter to lie on the dorsal cord. The exposed end of the tubing was secured with cyanoacrylate and the guide wire was gently pulled from the tubing. Spinal transections were confirmed by (1) inspecting the cord during the operation, (2) observing the behavior of the rats after recovery to ensure that they exhibited paralysis below the level of the forepaws and did not vocalize to the leg shock, and (3) examining the spinal cord post-mortem in a randomly selected subset of the animals.

### Behavioral apparatus

**Figure 1:**
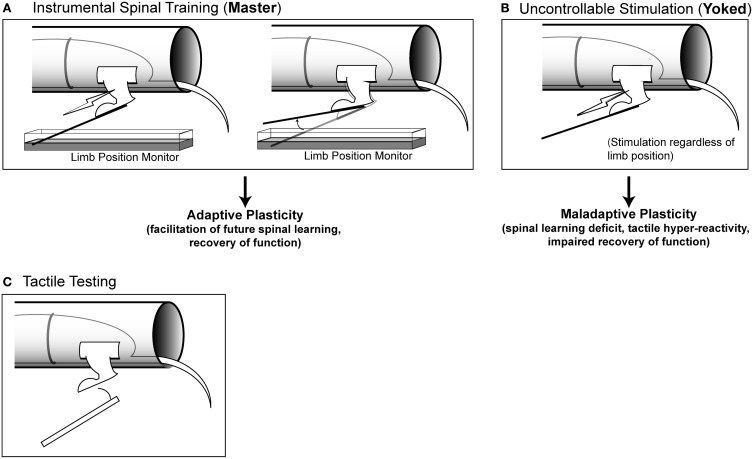
**Modes of stimulation and testing. (A)** Instrumental spinal training. Master rats receive stimulation to the tibialis anterior muscle of one hindlimb that is contingent upon leg position. When the limb is extended, stimulation is delivered, when the limb is flexed, stimulation is terminated. Master rats learn over time to keep the limb flexed to reduce stimulation exposure (spinal learning). **(B)** Uncontrollable stimulation. Yoked rats receive stimulation at the same time as their Master counterparts, regardless of limb position. These rats do not learn a response-outcome association and continue to fail when later tested with controllable stimulation. **(C)** Tactile testing. To assess hyper-reactivity, calibrated von Frey filaments are used to probe the ventral surface of the hindlimb. Filaments of increasing stiffness are used, until a response is made, and the force necessary to elicit a response is recorded.

We assessed spinal learning capacity as well as tactile responsiveness using a behavioral apparatus previously described (Grau et al., 1998; Ferguson et al., 2006; Figure 1). Briefly, spinally transected rats were placed in plexiglas tubes containing slots to allow the hindlimbs to hang freely. Rats were secured with an insulated wire belt that was gently wrapped around the rat and passed through holes on the side of the tube.

### Spinal learning paradigm

For spinal training studies, stainless steel leads from a BRS/LVE AC stimulator (Model SG-903; Laurel, MD, USA) were implanted into the tibialis anterior muscle, and the skin 1.5 cm above the ankle. Stimulation (60 Hz, constant current biphasic AC) intensity was initially set to 0.1 mA and then adjusted so that a single 0.3 s stimulus yielded a standardized flexion force of 0.4 N. Force was measured using strain gauge (Fort-1000; World Precision Instruments) attached to the foot with a monofilament plastic line (4-lb test; Stren, DuPont). The strain gauge output was fed through a multimeter calibrated to allow conversion between voltage and force in N. Instrumental (response-outcome) learning in the spinal cord was evaluated by arranging a relationship between leg position (response) and shock to the tibialis anterior muscle (outcome). Prior work has shown that in the presence of this controllable stimulation the spinal cord rapidly learns to hold the leg in a flexed position, minimizing shock exposure (Buerger and Fennessy, 1970; Grau et al., 1998; Jindrich et al., 2009). We assessed leg position by attaching a stainless steel contact electrode (7 cm × 0.46 mm) to the plantar surface of one hind leg and submerging the tip of the electrode 4 mm below the surface of an underlying saline solution. By placing a ground wire in the solution and attaching a fine wire to the contact electrode, we can monitor whether the leg is in an extended position, completing the circuit, or in a flexed position. The state of the circuit was monitored using an analog to digital converter (sample rate = 30 Hz) with digital outputs sent to a Macintosh computer. Stimulation was delivered each time the contact electrode touched the underlying solution. Stimulus onset occurred upon contact with the solution and stimulus offset occurred when the leg was lifted (minimum stimulus duration = 80 ms). Stimulation occurred for the duration of contact. Using this simple dichotomous measure of leg position (up vs. down) allows us to measure the time in the down position and the number of flexion responses. From these two measures we derive the mean response duration for each animal within 60 s time bins over the 30 min instrumental training period (Grau et al., 1998). Response duration for each animal was determined using the following formula:

$$\text{Response}\;\text{duration}=(60-\text{seconds}\;\text{in}\;{\text{solution}}_{i})/(\text{flexion}\;{\text{number}}_{i}+1),$$

where *i* is the current training bin.

### Master/yoked training procedures

The spinal cord was trained using a well-established three-group design consisting of master, yoked, and unshocked rats run simultaneously in sets of three rats (Figures 1A and 1B; Horridge, 1962; Buerger and Fennessy, 1970; Grau et al., 1998). All rats were prepared for spinal training as described above and then randomized to the three different conditions. Master rats received response-contingent stimulation: stimulation was delivered to the leg when it was in an extended position. Yoked rats received uncontrollable leg stimulation: the tibialis anterior was stimulated whenever their paired master extended the leg. Unshocked rats received no leg stimulation. Prior work has shown that under these conditions the master rats will acquire the flexion response, and yoked rats will fail (Horridge, 1962; Buerger and Fennessy, 1970; Grau et al., 1998; Jindrich et al., 2009). When all three groups are later re-tested with response-contingent stimulation, the master animals re-acquire the response at a faster rate than unshocked controls and the yoked animals fail to learn (Grau et al., 1998, 2004; Crown and Grau, 2001; Huie et al., 2012a). The learning deficit produced by yoked training represents a lasting form of maladaptive spinal plasticity that endures for >24 h in complete transection injuries, and produces long-term (>6 week) impairments in locomotor recovery after contusive SCI (Crown et al., 2002a; Grau et al., 2004).

### Intradermal formalin

To test for interactions between central sensitization and spinal training, we used the formalin test, a well-characterized central sensitization model from the pain literature (reviewed in Le Bars et al., 2001). Spinalized rats were given a single 50 μl subcutaneous injection of formalin in 1 of 4 concentrations (0, 5, 10, or 15%) in 0.9% saline into the dorsal surface of one hindpaw (in contrast to the plantar surface where tactile testing was performed). This manipulation produces a well-documented sensitization of spinal neurons (Coderre, 2001) that can be blocked by N-methyl-D-aspartate receptor (NMDAR) antagonists (Coderre and Melzack, 1992; Yamamoto and Yaksh, 1992).

### Tactile testing

**Figure 2:**
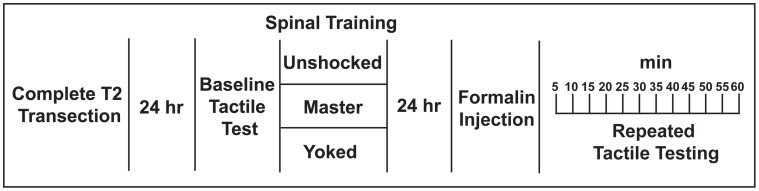
**Experimental design used to test whether spinal training history alters future nociceptive responsiveness in the formalin test**.

Hindpaw tactile testing was performed on spinalized rats placed in loose restraint tubes and secured as described the “Behavioral Apparatus” section (Figure 1C). After a 5 min acclimation period baseline tactile reactivity was established using von Frey stimuli (Semmes-Weinstein). Von Frey stimuli consisted of standardized polymer monofilaments of differing diameters that were delivered serially with increasing von Frey filament forces until the stimulation elicited a flexion response. When flexed against the skin, each filament delivers a standard force of a known intensity. All tactile reactivity testing was performed on the plantar surface of the hindpaw (in contrast to the dorsal surface where formalin was delivered). All tactile testing was performed by raters who were blind to experimental conditions. Baselines were established prior to spinal training (Figure 2). Baseline was established twice on both the ipsilateral (shocked) and contralateral leg in a counterbalanced ABBA order and then averages were produced for each leg. Rats were then given master/yoked/unshocked training to one leg. Experimenters who were blind experimental condition performed threshold testing using calibrated von Frey filaments on the ipsilateral and contralateral limb in a counterbalanced ABBA sequence every 5 min for 60 min post-injection. Re-testing of the same paw was spaced 2 min apart. Further details can be found in prior studies (Ferguson et al., 2006).

### Intrathecal NMDA delivery

Spinalized rats received intrathecal injections of NMDA (Tocris, Ellisville, MO) in 1 of 3 doses (1.0, 10.0, or 100 nmol) with a control of 15 μl 0.9% saline. The drug was delivered over the course of 3 min followed by a 10 μl saline flush over 2 min. Immediately after drug delivery rats were placed in Plexiglas tubes. Twenty-four hours later rats were prepared as described above under “Spinal Learning Paradigm” and tested with 30 min of response-contingent leg stimulation.

### Statistics

Results were analyzed using balanced experimental designs and mixed, factorial analyses of variance (ANOVA) by the GLM protocol in SPSS v.19 (IBM). The general statistical workflow adhered to highly-cited analytical standards (Keppel and Wickens, 2004). The statistical plan consisted of testing higher-order interactions that were intrinsic to the *a priori* experimental design, and then significant effects were distilled in waves of lower order interaction testing, and ultimately testing of main effects followed by Tukey's *post-hocs* on group means where appropriate. Significance was assessed at *p* < 0.05. All graphs reflect group means and error bars reflect standard error of the mean (SEM).

## Results

The experiments were designed to test for cross-talk between spinal training and nociceptive plasticity in complete SCI. The experiments are complementary to those reported elsewhere and interested readers are encouraged to examine complementary studies in Ferguson et al. (2006, 2008a) and Huie et al. (2012a). The present paper is also linked to companion reviews by Grau et al. (2012) and Ferguson et al. (2012) in the present issue of Frontiers in Integrative Physiology which provide further theoretical background for the present studies.

### Irritant-induced sensitization of tactile sensitivity below complete SCI

To test whether a nociceptive barrage could undermine normal spinal function below a complete SCI we used a well-established chemical irritant that directly drives nociceptors: intradermal formalin. Numerous papers have shown that a dilute formalin solution injected into the dorsal surface of the hindpaw strongly activates primary nociceptive afferents resulting in both peripheral and central sensitization (Hunskaar and Hole, 1987; Coderre and Melzack, 1992; Yamamoto and Yaksh, 1992; Le Bars et al., 2001; Yashpal et al., 2001). Behaviorally, the formalin test results in tactile hyper-reactivity as well as spontaneous nocifensive behaviors such as licking the paw, or hind limb guarding (Dubuisson and Dennis, 1977). In intact animals these nocifensive behaviors occur in two phases: an early phase (5 min), followed by a quiescent period and then re-emergence of a late phase (25–60 min). It has been argued that the different phases of formalin pain involve different mechanisms that may be organized at different anatomical levels. The early phase reflects hyperactivity in spinal nociceptive system that is then inhibited by segmental and descending brain stem pathways. The second phase is thought to reflect a brain-mediated change that alters descending control over spinal nociception, resulting in a secondary phase of hyper-reactivity (Abbadie et al., 1997; Xu et al., 2010). Accordingly, prior work has shown that complete spinal transection abolishes the late phase response whereas the early phase hyper-reflexia remains largely intact (Wheeler-Aceto and Cowan, 1991).

To confirm that formalin alters spinal nociceptive function in our complete transection model of SCI, (Grau et al., 1998), we performed a complete T2 transection by cautery and performed hindpaw formalin testing 48 h post-injury (Figure 2, unshocked group). Because complete transection abolishes supraspinal responses to hind-paw formalin, we limited our behavioral testing to tactile responses of the plantar hindpaw.

**Figure 3:**
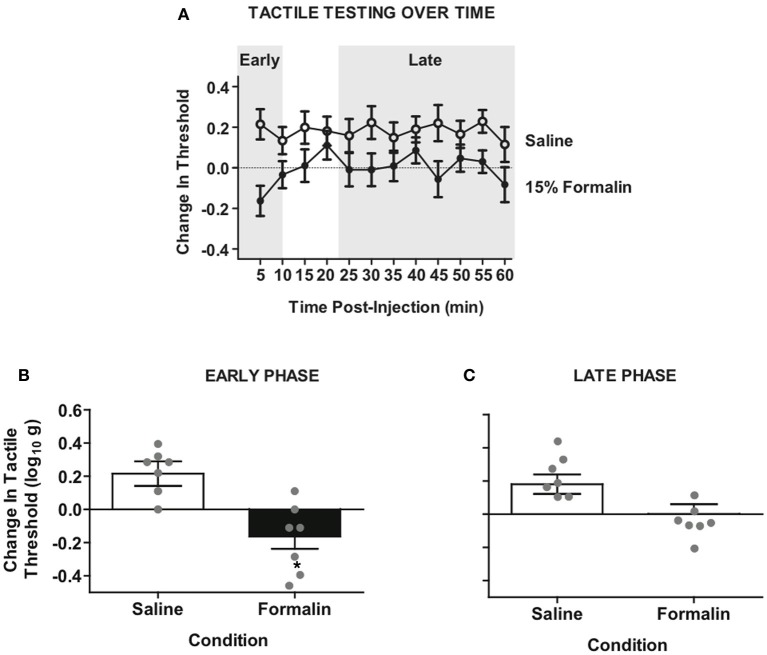
**Intradermal formalin produces ipsilateral hyper-reactivity in rats with complete SCI. (A)** Time-course of hyper-reactivity with repeated von Frey testing on the plantar surface of ipsilateral hindpaw after intradermal formalin injection. Mixed factorial Three-Way ANOVA revealed significant interaction of testing side × condition, *F*_(1, 12)_ = 5.59, *p* < 0.05. **(B)** Significant hyper-reactivity on the early phase formalin response ipsilateral to injection *F*_(1, 12)_ = 13.11, ^*^p < 0.01, *n* = 7 rats/formalin condition. **(C)** Non-significant trend of hyper-reactivity in the late phase response, *p* = 0.059. Points and bars represent group means (± SEM), gray points reflect the individual animals.

**Figure A1:**
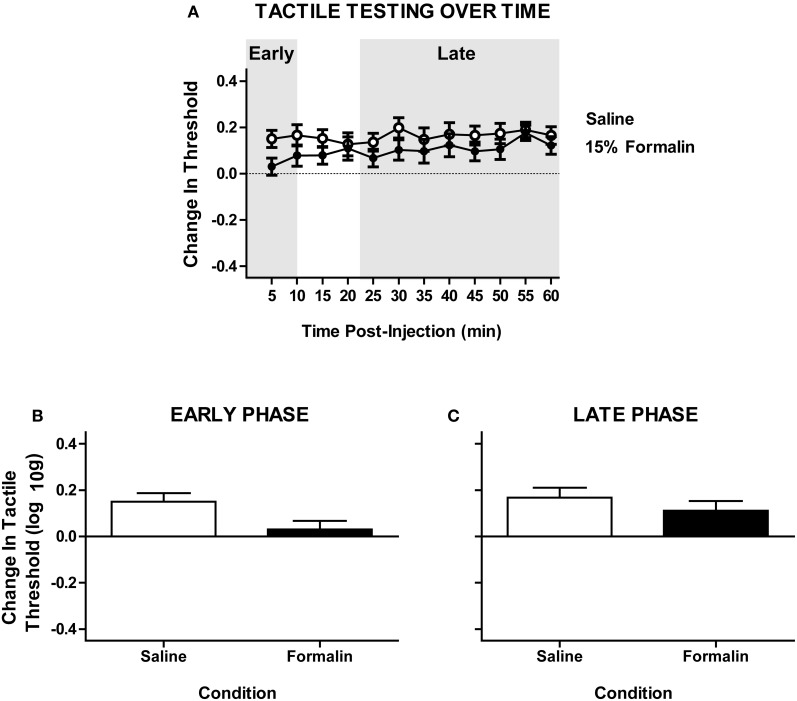
**Intradermal formalin does not produce a significant contralateral hyper-reactivity in untrained subjects with complete SCI. (A)** Time-course of tactile response with repeated von Frey testing on the plantar surface of ipsilateral hindpaw after intradermal formalin injection. **(B)** Non-significant trend toward hyper-reactivity on the early phase formalin response contralateral to injection [*F*_(1, 12)_ = 2.17, *p* = 0.166, *n* = 7] rats/formalin condition. **(C)** Lack of hyper-reactivity in the late phase response, *P* » 0.05. Points and bars represent group means (±SEM).

Formalin produced persistent tactile hyper-sensitivity on the ipsilateral hind paw relative to vehicle injection (Figure 3). This hyper-reactivity to formalin was significant on the ipsilateral leg (Figure 3A) but not the contralateral leg (Figure A1). Because the early phase (first 5 min) and the late phase (25–60 min) of the formalin response involve distinct mechanisms (Coderre and Melzack, 1992; Yamamoto and Yaksh, 1992), we next performed separate analyses of the different phases on the ipsilateral leg (Figures 3B,C). Analysis of the early phase confirmed a pronounced hyper-sensitivity (Figure 3B), however, the late phase response was diminished (Figure 3C). Together the data confirm that formalin produces a behaviorally discernible nociceptive response in our complete transection SCI model. It is noteworthy that the most pronounced sensitivity was observed on the ipsilateral leg and in the early phase of the formalin test, replicating previous observations in complete transection injuries (Wheeler-Aceto and Cowan, 1991).

### Spinal training history affects irritant response in complete SCI

**Figure 4:**
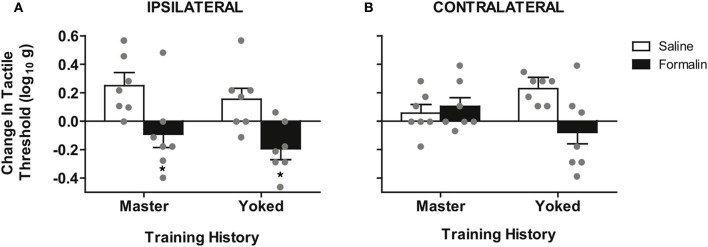
**Spinal training history alters formalin hyper-reactivity contralateral to the injection. (A)** Significant hyper-reactivity response to formalin but no differential effect of master/yoked training history on the ipsilateral leg, ^*^p < 0.05 from saline, *n* = 7 rats/group. There was also no significant difference between master/yoked/unshocked on ipsilateral hyper-reactivity, all *p* > 0.05, (compare to Figure 3B). **(B)** Significant enhancement of formalin hyper-reactivity in yoked group contralateral to injection, ^*^p < 0.05 from yoked saline, *n* = 7 rats/group. Bars represent group means (± SEM) gray points reflect the individual animals. Four-Way mixed, double repeated measures ANOVA was used for an integrative test of full experimental design including: training history (between subjects), formalin condition (between subjects), leg laterality (within subjects), time (within subjects). Modulatory effects of training history on formalin reactivity were re-affirmed by significant interaction of training history × formalin × time, (*p* < 0.05). The Four-Way interaction of leg laterality × training history × formalin × time did not reach significance, *p* > 0.05, reinforcing the bilateral (central) nature of training-enhanced nociception.

To test whether spinal cord training produces central changes that alter formalin nociceptive reactivity, we randomized a new group of rats into master/yoked training pairs at 24 h post-injury (24 h pre-formalin; for design see Figure 2). For each training pair, master rats received leg position-dependent stimulation and yoked animals received stimulation along with the master, irrespective of leg position. This experimental design ensures that master rats and yoked rats receive the same amount of leg stimulation, yet the master experiences stimulation that is dependent upon their leg position while the yoked experiences leg stimulation that is uncontrollable (Horridge, 1962; Buerger and Fennessy, 1970; Grau et al., 1998). We have previously found that uncontrollable stimulation of one leg produces a bilateral tactile hyper-sensitivity in complete SCI (Ferguson et al., 2006), and the same response is observed with direct activation of nociceptors using intradermal capsaicin (Hook et al., 2008). It has been argued that ipsilateral hyper-reactivity reflects both peripheral and central changes, whereas only contralateral changes provide a pure measure of central sensitization (Woolf, 1983; Milligan et al., 2003). For this reason we contrasted the effects of master/yoked training on formalin reactivity ipsilateral and contralateral to the injection. Laterality of the training leg was counterbalanced with respect to the injection, thereby controlling for peripheral effects of training history. As shown in Figure 4A, early phase formalin responses on the ipsilateral limb did not differ across master/yoked training groups, and both groups showed similar hyper-sensitivity as unshocked rats (compare with Figure 3B). The findings suggest that spinal training history does not alter peripheral sensitization in the formalin test.

To assess central sensitization we used the classic approach of testing tactile responsiveness on the contralateral leg (Woolf, 1983). This analysis revealed that yoked (uncontrollable stimulation) training enhanced future hyper-reactivity contralateral to formalin injection (Figure 4B). Specifically, yoked animals demonstrated a significant tactile hyper-reactivity on the contralateral limb that was not observed in either master or unshocked rats. In addition the modulatory effect of training history on formalin hyper-reactivity was not significantly different between ipsilateral and contralateral sides, confirming prior findings that training on one leg induces a central change that alters bilateral responsiveness. This is in contrast to the unilateral effects observed in the untrained (unshocked) rats shown in Figure 2. The bilateral nature of the training effects underscores that nociceptive hyper-reactivity produced by yoked training history is indeed a form of central sensitization (Grau et al., 1998; Crown et al., 2002b; Joynes et al., 2003; Ferguson et al., 2006; Baumbauer et al., 2008; Hook et al., 2008; Young et al., 2008; Huie et al., 2012a).

The findings strongly suggest that prior exposure to uncontrollable (yoked) stimulation in SCI rats primes them for later central sensitization by formalin injection. Notably the equivalent amount of response-specific instrumental (master) training did not sensitize the central nociceptive system.

### Formalin nociception produces impairment in spinal learning on the contralateral leg

**Figure 5:**
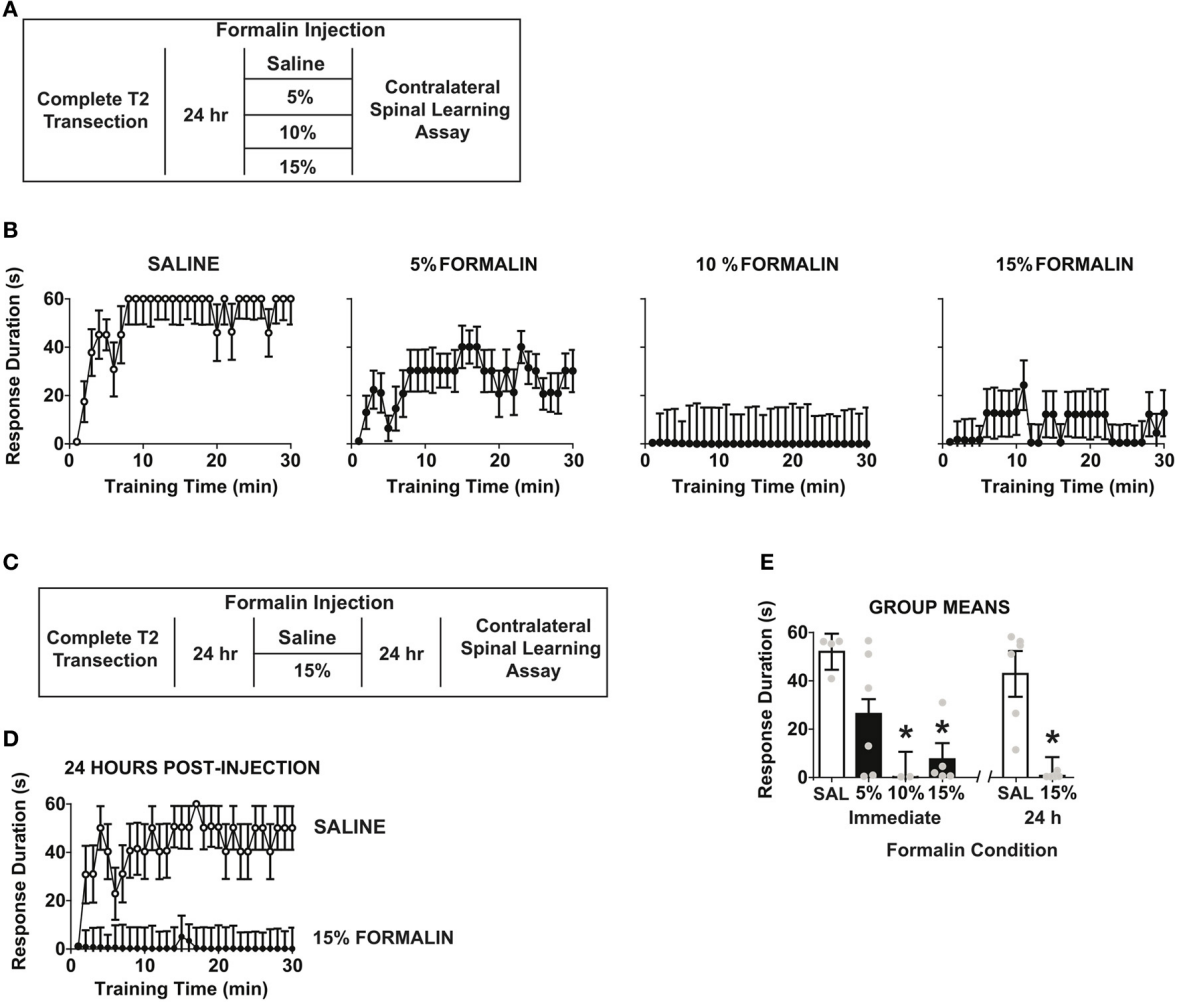
**Intradermal formalin produces contralateral impairments in spinal learning. (A)** Experimental design used to test immediate dose-response characteristics of formalin concentration on spinal learning potential. **(B)** Concentration-dependent impairment in spinal learning contralateral to formalin injection (0%, *n* = 4; 5%, *n* = 6; 10%, *n* = 2; 15%, *n* = 5; the numbers of subjects is supported by statistical power analysis, partial eta squared = 0.58, power = 0.89). Mixed repeated measures ANOVA revealed a significant effect of time, *F*_(29, 377)_ = 2.46, *p* < 0.05, and formalin concentration, *F*_(3, 13)_ = 6.15, *p* < 0.05. Tukey's *post-hoc* test revealed that 10% and 15% formalin significantly impaired spinal cord learning relative to 5% formalin and vehicle, *p* < 0.05. **(C)** Experimental design used to test effects of formalin on spinal learning potential. **(D)** Spinal learning impairment on the contralateral leg 24 h after formalin injection (*n* = 6 rats/group). Mixed repeated measures ANOVA revealed significant main effects of time, formalin, and time × formalin, all *p* < 0.05. **(E)** Group means for all formalin conditions, One-Way ANOVA confirmed effect of formalin condition *F*_(5, 23)_ = 8.64, *p* < 0.0001. *Post-hoc* Tukey's revealed saline groups did not differ, whereas 10, 15, and 15% 24 h formalin groups had significant learning impairments,^*^p < 0.05 from saline groups. Points and bars represent group means (± SEM), gray points reflect the individual animals.

The preceding experiments indicate that spinal training history influences the degree of central sensitization produced by formalin. We next tested the converse: whether formalin produces a central change that influences spinal cord learning in complete SCI. We performed a set of two independent experiments (Figures 5A,B). We first delivered formalin to the hind-paw at a range of concentrations after complete SCI and then assayed spinal learning on the contralateral leg. As shown in Figure 5C formalin produced a dose-dependent impairment of spinal learning on the contralateral limb.

To test whether formalin induced a lasting central change, we performed an independent study where we delivered 15% formalin or vehicle and then assayed spinal cord learning on the contralateral leg 24 h later. As shown in Figure 5D formalin produced a long-term deficit in spinal learning. To contrast the long-term and short-term effects of formalin we performed an additional analysis comparing all doses and post-injection time points. The findings revealed equivalent deficits in spinal learning in immediate and 24 h post-formalin conditions (Figure 5E).

The findings indicate that peripheral nociceptive activation with formalin induces a lasting central change in spinal cord learning in rats with complete SCI. Together with the prior findings the data suggest that the specific pattern of peripheral stimulation dictates the form of central plasticity that is invoked after injury, leading to either central sensitization or adaptive spinal cord learning, depending on stimulus type.

### Central activation of spinal NMDA receptors produces enduring spinal learning impairments in SCI animals

The preceding experiments demonstrated that chemical nociception with hindpaw formalin injection impairs spinal cord learning in complete SCI. The fact that this effect is observed contralateral to the irritant strongly suggests that a form of central sensitization is involved. Central sensitization by formalin is known to require activation of spinal NMDA receptors (Haley et al., 1990; Dickenson and Sullivan, 1991). In addition, direct intrathecal delivery of NMDA ligand (5–50 mM) sensitizes dorsal horn neurons and produces spontaneous nociceptive behaviors in intact rats (Raigorodsky and Urca, 1987; Dougherty et al., 1992; Bjorkman et al., 1994; Menendez et al., 1997; Alvarez-Vega et al., 2000; Sato et al., 2003; Kim et al., 2008; Roh et al., 2009).

**Figure 6:**
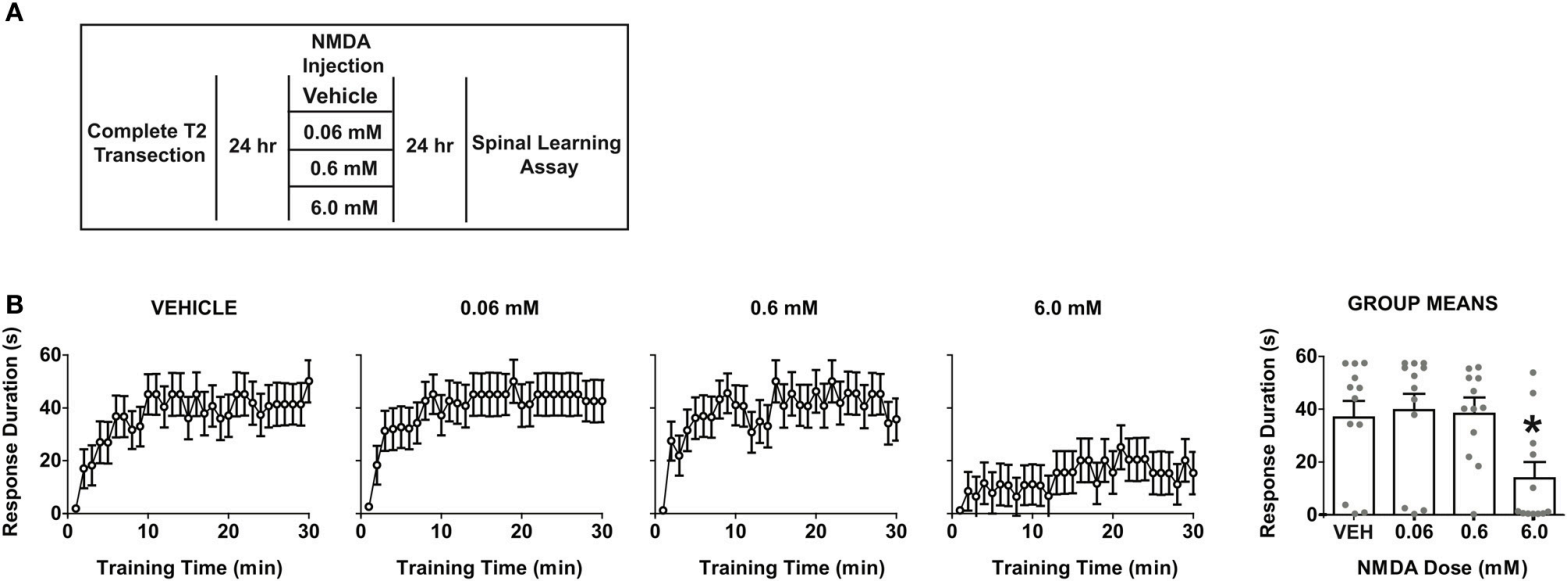
**Direct overactivation of spinal NMDA receptors impairs spinal learning 24 h later. (A)** Experimental design used to test the effect of intrathecal NMDA on spinal learning potential 24 h after delivery. **(B)** Dose-response function for NMDA-induced impairment of spinal learning. Mixed-repeated measures ANOVA revealed a significant effect of time *F*_(29, 1276)_ = 10.69, *p* < 0.0001 and NMDA dose *F*_(3, 44)_ = 4.08, *p* < 0.05. Tukey's *post-hoc* revealed that the 6 mM dose produced a significant impairment relative to the other doses, ^*^p < 0.05 from other doses (*N* = 12 subjects/dose) gray points reflect the individual animals.

To test whether NMDA-induced central sensitization impacts spinal cord learning, we delivered intrathecal NMDA at three doses (0.06, 0.6, or 6.0 mM) and assayed spinal learning 24 h later (Figure 6A). If NMDA produces a central sensitization that mimics the enduring effects of uncontrollable stimulation and formalin, then NMDA should alter spinal learning at this delayed time point. As shown in Figure 6B, NMDA produced a dose-dependent impairment in spinal learning when tested at 24 h post-injection. The most robust deficit was observed with 6 mM. Together the data indicate that central sensitization by NMDAR activation generates an impairment in spinal learning. The findings strongly suggest that direct central sensitization undermines spinal cord learning in the injured spinal cord.

It is important to note that the dose necessary to impair spinal learning was relatively high when compared to prior studies of spontaneous nociceptive behaviors in intact animals, which have typically used doses ranging from 0.1 to 1 mM. This raises the possibility that the current effects could be due to an NMDA-induced excitotoxicity, which has been observed in primary cell culture at doses as low as 1 mM (Koh and Choi, 1988; Dawson et al., 1993). However, several lines of data suggest that our 6 mM intrathecal dose still falls within a normal physiological range *in vivo*. Studies of the locomotor central pattern generator (Grillner et al., 1981) have used intrathecal doses five times higher than our highest dose to elicit fictive locomotion in *in vivo* spinal rat preparations (Giroux et al., 2001). In addition studies in primate models have shown central sensitization of nociceptive responses with intrathecal NMDA doses of up to 50 mM. Together, this suggests our doses fall within a reasonable range for central sensitization and the learning deficits observed are more consistent with maladaptive plasticity than a wholesale excitotoxic lesion (Villanueva et al., 2002; Yoon et al., 2010). In combination with the other experimental findings and prior literature the NMDA findings reinforce the concept that central sensitization impairs adaptive spinal cord learning.

## Discussion

Prior literature and the new experimental findings presented here reinforce the concept that nociceptive plasticity opposes spinal learning adaptations after complete SCI. The results indicate that spinal training history can influence future nociceptive responsiveness in the formalin test. In particular pre-training with inappropriate uncontrollable (yoked) stimulation, but not instrumental (master) training, enhances later tactile hyper-reactivity produced by intradermal formalin into the hind paw. This training-enhanced reactivity was evident on the contralateral paw, strongly suggesting a central mechanism. Follow up experiments revealed that intradermal formalin, delivered at doses that enhance tactile reactivity, disrupts future spinal learning on the contralateral paw for at least 24 h. Finally, directly driving central NMDA receptors, which produces central nociceptive sensitization, generates a lasting (>24 h) impairment in spinal learning. Taken together the findings indicate that central sensitization and adaptive spinal learning are opposing forms of spinal plasticity.

The existing literature indicates many similarities between central sensitization within spinal pain pathways and stimulus-induced maladaptive spinal plasticity that prevents future spinal learning after SCI. As with many other forms of plasticity, both central sensitization and the spinal learning deficit are mediated by changes in glutamatergic activity. The NMDA receptor antagonist MK-801 has been used to block central sensitization, as well as to inhibit the induction of the spinal learning deficit induced by uncontrollable stimulation (Woolf and Thompson, 1991; Ferguson et al., 2006). Further, group I metabotropic glutamate receptors (mGluR1 and mGluR5) have been shown to be necessary for formalin-induced nociception, as well playing a key role in the development of chronic pain following spinal contusion injury (Fisher and Coderre, 1996; Mills et al., 2002). We have previously shown that these same mGluR subtypes are also necessary in order for uncontrollable stimulation to produce a spinal learning deficit. Taken together with the current experiments demonstrating formalin-induced undermining of adaptive spinal learning, these findings provide further behavioral evidence that central sensitization and the maladaptive spinal plasticity that inhibits spinal learning engage common central mechanisms.

It should be noted that the main focus of the work with this spinal learning model has been behavioral and pharmacological, rather than strictly physiological. Historically, those studying spinal plasticity with this model have focused on the central neurochemical factors that mediate the behavioral effects, while the physiological circuitry that is engaged by stimulation of the tibialis anterior muscle has been investigated less extensively. Our goal is to understand the training potential of the spinal cord and all of our experiments are designed to rule out peripheral effects by testing all effects on the contralateral limb. The current study evaluated the potential for established nociceptive stimuli (formalin and TA stimulation) to alter the future capacity for spinal cord training of a flexion response on the contralateral limb. Similarly, our evidence for central nociceptive plasticity in the current study has been provided by behavioral outcomes on the contralateral limb, rather than direct testing of fiber activation. Although we have previously found that these behavioral effects depend on stimulation of c-fibers, direct electrophysiological confirmation for the recruitment of fiber types and muscle group activation following stimulation of the tibialis anterior represents an important area for further research.

An improved understanding of the fiber types could also potentially help explain some of the formalin dose-response features observed in the present study. We found that the most profound learning deficits were observed at a relatively high dosing range (5–15% formalin). Recent work suggests that lower doses (0.5%) of formalin selectively engage primary nociceptors that express transient receptor potential (TRP) channel subtype TRPA1, whereas increasing doses (2–5%) engage a wider range that includes non-TRPA1 nociceptors (Braiz and Bausbaum, 2010). Based on the dose-response function in the present study, one might hypothesize that spinal cord learning is more impaired when a broad range of different nociceptive fiber populations are engaged. However, the relative role of different nociceptive populations remains an open question for further research.

Much of the work to understand the neurobiology of our observed behavioral effects has come from pharmacological manipulation of known mediators of plasticity and nociception. Several nociception-inducing substances have been implicated in spinal learning deficits in our model including substance P, capsaicin, carrageenan, intrathecal glutamate agonists, and intrathecal delivery of the cytokine tumor necrosis factor alpha (TNFα) (Ferguson et al., 2006, 2008a,b; Baumbauer et al., 2007; Hook et al., 2008; Huie et al., 2012b) Our recent interest in TNFα as a modulator of spinal learning provides additional links between spinal cord learning impairments and nociception. Findings from the pain literature indicate that TNFα is a potent mediator of nociceptive plasticity (Czeschik et al., 2008; Choi et al., 2010; Park et al., 2011; Zhang et al., 2011). TNFα is known to modulate synaptic strength by acting to increase glutamatergic signaling, and we have demonstrated this effect to play a role in excitotoxicity following SCI (Beattie et al., 2002; Ferguson et al., 2008b). We have recently shown that blocking TNFα activity prior to uncontrollable stimulation protects against maladaptive spinal plasticity, and that administration of exogenous TNFα is sufficient to undermine adaptive spinal learning (Huie et al., 2012b). Beyond a protective effect, inhibition of TNFα activity can also restore adaptive plasticity after stimulation-induced maladaptive plasticity has been induced (Huie et al., 2012b). The use of TNFα inhibitors after SCI to aid in recovery is showing promise, and these recent findings suggest that TNFα may work to improve recovery of sensory and locomotor function not only by attenuating nociception and excitotoxicity, but perhaps by rescuing the capacity for adaptive plasticity in the injured spinal cord (Genovese et al., 2006; Ferguson et al., 2008b; Marchand et al., 2009). Together these data provide a potential molecular mechanism for observed commonalities between spinal learning deficits and nociceptive sensitization. Further work is needed to provide further mechanistic support.

It is important to recognize the potential negative consequences of inappropriate training and nociceptive input for individuals with SCI. Even in complete SCI where a patient does not consciously experience pain below an injury, nociceptive input to the spinal cord may promote a maladaptive plasticity that undermines future spinal cord training and rehabilitation potential. Perhaps even more troubling, the present findings and prior studies suggest that inappropriate training of the spinal cord can generate a lasting central sensitization of nociceptive reactivity. Caudle et al. recently demonstrated that passive stretch therapy in rats after SCI hindered locomotor recovery, as did limb immobilization in a wheelchair (Caudle et al., 2011). Likewise, Petruska et al. found that in step-trained transected rats, the introduction of noxious stimuli (due to cutaneous hindpaw lesions) undermined the beneficial locomotor effects of step-training (Petruska et al., 2007). Thus, it is possible that impairment of recovery after SCI may reflect nociceptive plasticity induced by the uncontrollable afferent input, arising from numerous possible sources. Care must be taken in designing rehabilitative strategies for SCI patients to avoid therapies that might produce uncontrolled nociceptive input. One must be aware of the injured spinal cord's vulnerability to central sensitization, so that rehabilitation strategies that are intended to promote recovery do not end up creating new problems. Central sensitization can lay down an enduring pain memory in the spinal cord that interferes with the re-acquisition of adaptive behavior and fosters the development of neuropathic pain (Woolf, 1983). The supraspinal experience of neuropathic pain could be unveiled if future regenerative therapies successfully reconnect the brain to a lumbar spinal cord containing sensitized nociceptive circuitry. Further research is needed to ensure therapies that promote adaptive spinal plasticity while limiting central pain.

### Conflict of interest statement

Dr. Eric D. Crown is currently employed by Abbott Laboratories. The current research is not affiliated with his current duties at Abbott Laboratories. The other authors declare that the research was conducted in the absence of any commercial or financial relationships that could be construed as a potential conflict of interest.
